# A longitudinal analysis of the U.S. Air Force reserve officers’ training corps physical fitness assessment

**DOI:** 10.1186/s40779-019-0219-4

**Published:** 2019-09-23

**Authors:** Cameron S. Mackey, Jason M. DeFreitas

**Affiliations:** Applied Neuromuscular Physiology Laboratory, Oklahoma State University, 192 CRC, Stillwater, OK 74078 USA

**Keywords:** Class ranks, Fitness assessment, Military, Physical training, ROTC

## Abstract

**Background:**

The U.S. Air Force physical fitness assessment (PFA) is used to determine the overall fitness of their personnel. It is currently unknown to what extent the PFA scores of Reserve Officers’ Training Corps (ROTC) cadets are affected by mandatory physical training. The purpose of this investigation was to longitudinally examine the PFAs of ROTC cadets over a four-year period, evaluate the results across class ranks, and evaluate the sensitivity of the classification of the tests.

**Methods:**

Air Force ROTC cadets performed the PFAs (abdominal circumference, 1-min pushups, 1-min sit-ups, and a 1.5-mile run) in both the spring (*n =* 26) and fall (*n =* 22) semesters. PFAs were compiled over a four-year period (Spring 2014 – Fall 2017) and were performed in accordance with Air Force Instruction 36–2905. A one-way repeated measures ANOVA was performed separately for the fall and spring groups for each dependent variable across the 4 years. Additionally, a one-way between groups ANOVA was performed for each dependent variable during the time point (fall 2015; *N =* 46) with the most recorded cadets for each class rank.

**Results:**

Longitudinal assessments revealed a main effect of time (*P =* 0.010) on abdominal circumference; cadets had a smaller abdominal circumference in their freshman year than in their senior year. A main effect of time (*P =* 0.006) was also observed on sit-up quantity; cadets performed more sit-ups in their junior year than in their freshman year. Examining between class ranks during the same year (between-subjects ANOVA) revealed a main effect of class rank on sit-up quantity (*P =* 0.003); the freshmen completed fewer repetitions than the sophomores (*P =* 0.018) and the juniors did (*P =* 0.001).

**Conclusion:**

The results indicated that only the sit-up component showed differences between class ranks. These findings suggest that the Air Force PFA may not be sensitive enough to detect changes in physical fitness or distinguish between class ranks regarding physical performance, even after years of training. This limitation may be in part due to the limited duration of training incorporated by the ROTC program (2 h per week), which provided a maintenance effect rather than improvement in physical performance. We recommend that more attention be directed to the efficacy of physical training, the sensitivity of measures included in the PFA, or both.

## Background

Physical fitness is important for general health and the ability to perform activities of daily living and occupational tasks. In military services, higher levels of physical fitness are vital for limiting modifiable risk factors (e.g., obesity and risk of injury), improving military task-specific performance, and preventing injuries. Thus, sufficient levels of physical fitness are emphasized in military personnel due to the high physical demands during military training and in warfare [1]. In a military context, the term “physical fitness” is predominantly identified as muscular strength, muscular endurance, cardio-respiratory endurance, and body composition [2]. However, the importance of each of these components may vary between different types or branches of services. For example, the U.S. Army physical fitness components (a 2-mile run, 2 min of pushups, and 2 min of sit-ups) are worth up to 100 points each; the scores of each component are summed together and then compared to an overall score of 300 [3]. Currently, the U.S. Air Force uses Air Force Instruction (AFI) 36–2905 to conduct its fitness test, which consists of 1 min of pushups, 1 min of sit-ups, an abdominal circumference measurement (inches), and a 1.5-mile run [4]. However, unlike the U.S. Army, the U.S. Air Force does not weight each component equally. Rather, an airman receives up to 60 points for the run, 20 points for the abdominal circumference measurement, and 10 points each for the sit-up and pushup components.

The U.S. Air Force Reserve Officers’ Training Corps (ROTC) program is designed to recruit, educate and commission officer candidates through college campus programs based on the U.S. Air Force requirements. The ROTC program prepares the cadets to become U.S. Air Force officers while earning a college degree. At this time, the ROTC acts as the largest commissioning source among all military branches, and the Air Force ROTC program is offered at more than 1100 colleges and universities across the U.S. To join the ROTC during college, students enroll in in the ROTC course as they would enroll in any other course. Throughout the program, the students must adhere to the ROTC rules/guidelines and pass the physical fitness assessments each semester. Students can add/drop the ROTC program at any time. However, after students’ second year in the program, they must be selected to complete field training. Upon completion of field training, cadets are then offered a contract for a stipend during their final 2 years and commission once they have finished the program. U.S. Air Force ROTC programs are designed to physically prepare cadets for the demands of a military career. As a result, an important element of the ROTC experience is physical training (PT), which is meant to enhance physical fitness, develop discipline, and provide a unifying experience [5]. Specifically, PT is incorporated as a part of the U.S. Air Force culture to establish an environment for members to maintain physical fitness and health and meet expeditionary mission requirements [4]. For this reason, an effective assessment of physical fitness is necessary to determine if the PT has resulted in improvements over the duration of the ROTC program [6]. Currently, U.S. Air Force ROTC cadets are required to participate in PT two times per week. These mandatory PT sessions consist of a warm-up (stretching and calisthenics), upper- and lower-body strengthening exercises, and running. While there have been numerous studies investigating the physical fitness of U.S. Army ROTC cadets [5, 7–13], there is a scarcity of research regarding the U.S. Air Force ROTC population. Therefore, the purpose of this investigation was to longitudinally examine U.S. Air Force ROTC cadets over a four-year period for the evaluation of potential differences between class rank within the ROTC utilizing the current physical fitness assessment (PFA) and for the evaluation of the sensitivity of the classification of the tests in terms of absolute test results and composite scores. We hypothesized that PFA performance scores would increase with years in the program (i.e., more PT and experience with the PFA).

## Methods

### Experimental procedure

Collected data were gathered for analysis from the university U.S. Air Force ROTC program. PFA scores were recorded from two separate classes, one starting in the spring of 2014 and the other starting in the fall of 2014, and then the scores were compiled over a four-year period. The fall and spring groups were then measured/tracked across all 2 years to assess whether the PFA scores improved during the students’ time in the ROTC program. In addition, the time point at which the largest number of cadets took the PFA [fall 2015; *N =* 46; freshman (*n =* 8) vs. sophomore (*n =* 12) vs. junior (*n =* 17) vs. senior (*n =* 9)] was examined to evaluate potential differences between class ranks.

### Subjects

Male U.S. Air Force ROTC cadets from the spring (*n =* 26) and fall (*n =* 22) classes (mean ± SD: age 19.8 ± 1.2 years; height 178.1 ± 5.4 cm; weight 74.9 ± 7.7 kg; body mass index 23.4 ± 2.0) performed the PFA (body composition evaluation, 1 min of pushups, 1 min of sit-ups, and a 1.5-mile run) each year for four years. Only the cadets who completed all four years of the program were included in the analysis. Cadets participated in mandatory PT sessions twice per week, which generally consisted of a warm-up, pushups, pull-ups, sit-ups, and running. The university institutional review board for human subject research approved this study. This study was exempt from the consent process because the data included in this study were previously archived (i.e., retroactive) and deidentified (ED-18-39 STW IRB).

### Procedures – U.S. Air Force physical fitness assessment

The officers and noncommissioned officers conducted the field-based tests according to Air Force Instruction 36–2905 as part of the usual program assessment practices [4]. Body composition was the first component assessed, followed by the timed pushups, sit-ups, and 1.5-mile run. A standard rest period of 3 min was enforced between components.

### Body composition

Body composition included height, weight, and abdominal circumference measurements (inches). However, only the abdominal circumference measurement was used for the body composition component score. For each measurement, the cadets stood stationary while the tester conducted the measurement; the tester started at the superior border of the iliac crest of a cadet and moved around the cadet to place the tape in a horizontal plane around his or her abdomen. The tester took three measurements, and the average of the measurements was recorded for the abdominal circumference score. Cadets remained in the Air Force physical training attire (t-shirt, shorts and/or pants) for the duration of the body composition assessments.

### Timed pushups

Cadets performed pushups starting in the “up” position, in which the hands were placed slightly wider than shoulder-width, the palms or fists were placed on the floor, the arms were fully extended, and the rigid hip and spinal posture was maintained. On command, the cadets would bend their elbows and lower their entire body as a single unit until their upper arms were at least parallel with the ground (elbows bent at 90 degrees). The cadets returned to the starting position by raising their entire body until their elbows were fully extended. Any deviation from this form by a cadet resulted in the attempt not being counted toward the cadet’s component score. Cadets performed continuous pushups for 1 min, and the results were recorded.

### Timed sit-ups

Cadets performed sit-ups by starting on their back with their knees bent at a 90-degree angle and their feet or heels in contact with the floor. A partner held the cadet’s feet with his or her hands, applying adequate pressure across the dorsum of the foot to keep the heels anchored to the floor. The heels were required to remain in contact with the ground throughout the test. With their arms crossed over their chest and their hands/fingers placed on their shoulders or upper chest, cadets performed a complete repetition when they rose from the down position (i.e., upper torso is raised from the floor/mat) until their elbows touched their knees or thighs and then returned to the down position so that their shoulder blades touched the floor/mat. Any deviation from this form resulted in the attempt not being counted. Cadets performed continuous sit-ups for 1 min, and the results were recorded.

### 1.5-mile run

Cadets gathered at a 400-m track and were briefed about the purpose and organization of the test. An officer or noncommissioned officer then used a stopwatch to time each cadet as he completed the 1.5-mile run. The total time (s) was recorded. A standardized set of instructions was read to the cadets [4]:

“This 1.5 mile timed run is used to measure cardio-respiratory fitness. Prior to beginning the 1.5 mile run, you may complete up to a 3-minute warm-up. You will line up behind the starting line and will be instructed to begin running as I start the stopwatch. No physical assistance from anyone or anything is permitted. Pacing is permitted if there is no physical contact and is not a hindrance to other runners. You are required to stay on and complete the entire marked course. Leaving the course is disqualifying and terminates the test. Your completion time will be recorded when you cross the finish line and you are required to complete a cool down for approximately 5 minutes. If at any time you are feeling in poor health, you are to stop running immediately and you will be given assistance.”

### Statistical analyses

One-way repeated measures ANOVA was performed separately for the fall (*n =* 22) and spring (*n =* 26) groups for each dependent variable (pushups, sit-ups, abdominal circumference, run time, and composite score) across the 4 years. One-way between groups ANOVA was performed for each dependent variable during the time point (fall 2015; *N =* 46) with the most recorded cadets for each class rank [freshman (*n =* 8) vs. sophomore (*n =* 12) vs. junior (*n =* 17) vs. senior (*n =* 9)]. When sphericity was violated, Greenhouse-Geisser results were reported. Partial eta squared (${\eta}_p^2$) values were reported to estimate ANOVA effect sizes. PASW software (version 23.0, SPSS Inc., Chicago, IL, USA) was used for all statistical analyses. An alpha level of *P* ≤ 0.05 was considered significant for all comparisons.

## Results

### Longitudinal assessment

**Fig. 1:**
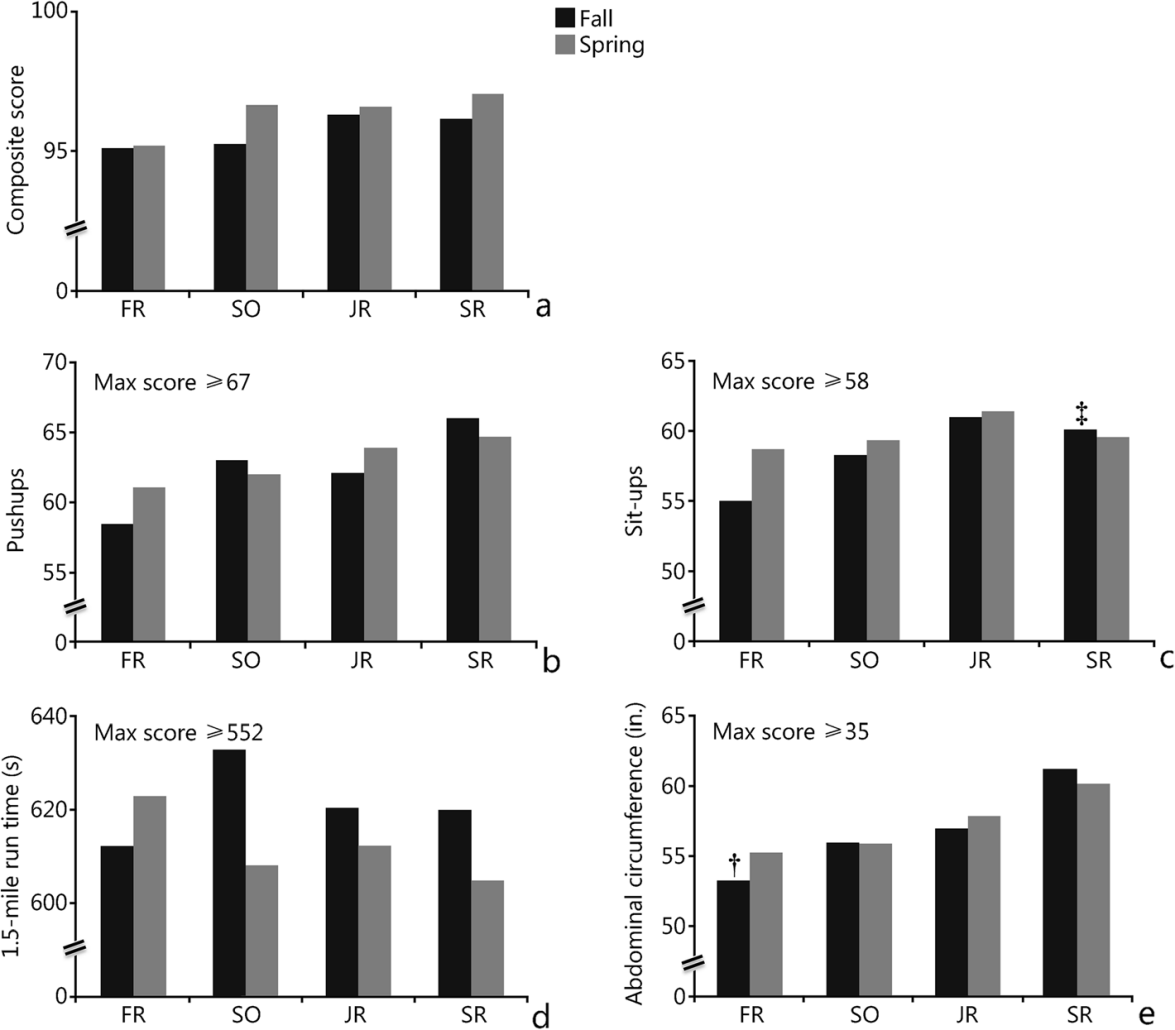
Longitudinal assessment of the freshman (FR), sophomore (SO), junior (JR), and senior (SR) class ranks of two classes (fall class = black; spring class = gray) over 4 years; the figures are separated by each component of the U.S. Air Force physical fitness assessment. Maximum scores are labeled according to AFI 36–2905. †. Significantly different from senior year (*P =* 0.026). ‡ . Significantly different from freshman year (*P =* 0.006)

For the fall semester measurements (Table 1; Fig. 1), there was a significant main effect of time (*P =* 0.010, ${\eta}_p^2$ = 0.187) on abdominal circumference; the cadets had a smaller measurement in their freshman year [mean (M) = 31.64, standard error of mean (SEM) = 0.425] than in their senior year (M = 33.23, SEM = 0.306; *P =* 0.026). In addition, a significant main effect of time (*P =* 0.006, ${\eta}_p^2$ = 0.180) on sit-ups was observed; students in their junior year (M = 60.96, SEM = 0.979) completed significantly more sit-ups than students in their freshman year (M = 54.96, SEM = 1.459; *P =* 0.006). However, no significant main effect of time was observed on pushups (*P =* 0.076, ${\eta}_p^2$ = 0.112), run time (*P =* 0.665, ${\eta}_p^2$ = 0.021), or the composite score (*P =* 0.73, ${\eta}_p^2$ = 0.020). For the spring semester measurements (Table 2; Fig. 1), no significant main effect of time was observed on abdominal circumference (*P =* 0.188, ${\eta}_p^2$ = 0.064), pushups (*P =* 0.458, ${\eta}_p^2$ = 0.034), sit-ups (*P =* 0.261, ${\eta}_p^2$ = 0.052), run time (*P =* 0.659, ${\eta}_p^2$ = 0.017), or the composite score (*P =* 0.263, ${\eta}_p^2$ = 0.052).

**Table 1:** Longitudinal assessments (mean ± SD) tracking Class 2 from fall 2014 to fall 2017 (4 years) in ROTC training

Class rank	Year	*n*	Ab. Circ. (in.)	Sit-ups (reps)	Pushups (reps)	Run time (s)	Total score
FR	Fall 2014	22	31.6 ± 2.0*	55.0 ± 6.8	58.5 ± 9.7	612.3 ± 62.4	95.1 ± 4.6
SO	Fall 2015	22	32.2 ± 1.9	58.2 ± 4.8	63.0 ± 9.7	633.1 ± 64.6	95.3 ± 4.1
JR	Fall 2016	22	32.4 ± 1.8	61.0 ± 4.6†	62.1 ± 10.8	620.6 ± 55.9	96.3 ± 3.7
SR	Fall 2017	22	33.2 ± 1.4	60.1 ± 6.6	66.0 ± 7.3	619.9 ± 72.2	96.2 ± 5.2

*Ab. Circ* Abdominal circumference measurement, *in* Inches, *FR* Freshmen, *SO* Sophomores, *JR* Juniors, *SR* Seniors. *. Significantly smaller than SR year (*P =* 0.026); †. Significantly more reps than FR year (*P =* 0.006)

**Table 2:** Longitudinal assessments (mean ± SD) tracking Class 1 from spring 2014 to spring 2017 (4 years) in ROTC training

Class rank	Year	*n*	Ab. Circ. (in.)	Sit-ups (reps)	Pushups (reps)	Run time (s)	Total score
FR	Spring 2014	26	32.0 ± 2.0	58.6 ± 4.6	61.0 ± 11.4	622.9 ± 73.6	95.2 ± 4.7
SO	Spring 2015	26	32.2 ± 1.8	59.2 ± 5.3	62.0 ± 10.3	608.0 ± 60.7	96.6 ± 3.6
JR	Spring 2016	26	32.6 ± 1.6	61.4 ± 6.7	64.0 ± 10.2	612.2 ± 60.4	96.6 ± 3.3
SR	Spring 2017	26	33.0 ± 1.7	59.5 ± 5.0	64.7 ± 7.1	605.0 ± 53.4	97.1 ± 3.3

*Ab*. *Circ* Abdominal circumference measurement, *in* Inches, *FR* Freshmen, *SO* Sophomores, *JR* Juniors, *SR* Seniors

### Between-subjects assessment

**Fig. 2:**
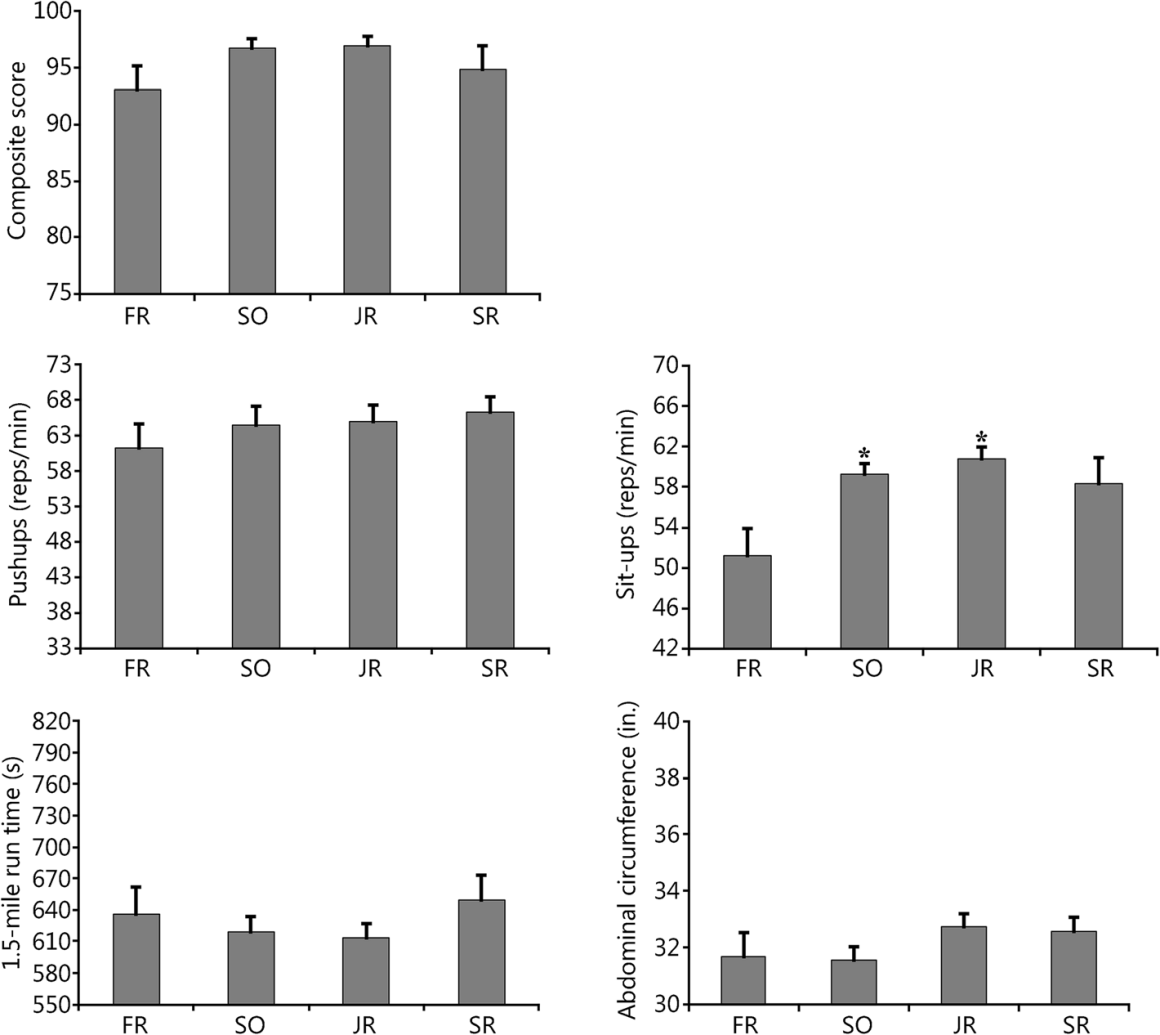
Between-subjects assessment (fall 2015 classes compared between each other) between freshman (FR), sophomore (SO), junior (JR), and senior (SR) class ranks for each component of the U.S. Air Force physical fitness assessment. *. Significantly different from freshman year (*P =* 0.001)

The between-subjects ANOVA (across classes at the same time point; fall 2015) revealed a significant main effect of class rank on sit-ups (*P =* 0.003); the freshman class (M = 51.25, SEM = 2.63) completed significantly fewer repetitions than both the sophomore class (M = 59.25, SEM =1.01; *P =* 0.018) and the junior class (M = 60.82, SEM = 1.09; *P =* 0.001) did. However, no significant main effects of class rank were observed on abdominal circumference (*P =* 0.286), pushups (*P =* 0.723), run time (*P =* 0.486), or the composite score (*P =* 0.210; Table 3; Fig. 2).

**Table 3:** Between-subjects assessment (mean ± SD) across classes during the fall semester in 2015 in which the largest number of cadets took the physical fitness assessment

Class rank	*n*	Ab. Circ. (in.)	Sit-ups (reps)	Pushups (reps)	Run time (s)	Total score
FR	8	31.7 ± 2.3	51.3 ± 7.4	61.3 ± 9.7	635.8 ± 74.4	93.0 ± 5.9
SO	12	31.6 ± 1.6	59.3 ± 3.5†	64.4 ± 9.6	618.8 ± 50.0	96.7 ± 3.1
JR	17	32.8 ± 1.9	60.8 ± 4.5†	64.9 ± 9.9	613.7 ± 54.5	96.9 ± 3.6
SR	9	32.6 ± 1.5	58.3 ± 7.6	66.2 ± 6.7	649.7 ± 69.0	94.8 ± 6.2

*Ab. Circ* Abdominal circumference measurement, *in* Inches, *FR* Freshmen, *SO* Sophomores, *JR* Juniors, *SR* Seniors. †. Significantly more reps than FR class (−0.018 ≤ *P* ≤ 0.003)

## Discussion

The primary finding of the present investigation was a lack of improvement in most of the variables measured during the longitudinal assessment. These findings raise into question whether the U.S. Air Force PFA is sufficiently sensitive to identify physical training-induced changes or the effectiveness of the current PT regimen. Specifically, this result could be due to how PT is administered (i.e., only training toward the testing standards). However, the fact that these PFAs generally took place later in the semester (i.e., fall testing occurred in November and spring testing occurred in April) may also be a factor. A study performed by Crawley et al. [13] lends support to this notion, in which the authors found that throughout a 16-week training program with police academy cadets, the cadets showed significant improvements in physical fitness characteristics in only the first 8 weeks. None of the variables they assessed (hand grip, arm crank, Wingate, body composition, 40-yard dash, 1-repetition maximum bench press, agility T-test, and sit-and-reach) showed a significant change across the second half of the program. Consequently, the authors indicated that modifications to training programs should be made to increase the overall effectiveness of PT, specifically after the 8-week period. Thus, it is realistic to assume that if similar or repetitive PT sessions are being implemented twice a week during the ROTC program, then significant improvements may not occur in the PFA components measured late in the semester (i.e., after 8 weeks of PT).

While the fall semester measures revealed significant differences in abdominal circumference between the freshmen and seniors (i.e., freshman had a smaller abdominal circumference than the seniors) and in sit-ups between the freshmen and juniors (i.e., the juniors performed more sit-ups than the freshmen), no significant differences were observed across the spring semester testing sessions. These findings may be a result of the required PT improving the PFA scores and overall fitness of the freshmen to the PFA and fitness levels of their fellow upperclassmen cadets. Recently, Oliver et al. [14] investigated the effects of PT in freshman U.S. Army ROTC cadets throughout the 9-month academic year. Their results indicated that PT is effective in improving the fitness level of freshman cadets when implementing the Army PFA. Interestingly, the improvements were evident when the PFA was used, while only minor improvements in performance were observed when clinical-based measurements were used. Specifically, no significant changes in body composition, maximal aerobic capacity, or lower-body power were observed. Additionally, while there were significant differences in the cadets’ scores from the pretraining to mid-training evaluations, no significant differences occurred between mid-training to posttraining evaluations for the male cadets, which is in support of our findings as well as the findings of the aforementioned study by Crawley et al. [13]. However, it is important to note that the U.S. Air Force ROTC cadets who participated in the present investigation scored exceptionally high in each of the PFA components as well as the composite score (Table 4). Due to these observations, it is possible that our findings showed few improvements as the cadets progressed in the program in addition to few differences between class ranks. Another reason for the small differences may be linked to cadet attrition rates during the program (~ 30 cadets drop per academic year). While the exact reasons that the cadets dropped the program are not known, some of the cadets may have dropped in part due to an inability to sustain a physical level matching that of their fellow cadets. Consequently, it may be beneficial for the mandatory physical training to be adjusted for cadets who are just entering the program or are less fit to improve their physical performance, increase retention rates, and better prepare them for their future military career.

**Table 4:** Means and standard deviations of Air Force ROTC cadets collapsed across class ranks for each component of the physical fitness assessment and the points achieved for each component as well as the minimum and maximum point thresholds for each component

Item	Ab. Circ. (in.)	Pushups	Sit-ups	Run time (s)	Composite score
Cadets	32.4 ± 1.8	62.6 ± 9.6	59.1 ± 5.5	616.7 ± 62.9	–
Points awarded	20.0	8.7–10.0	9.2–10.0	54.8–59.7	96.0 ± 4.1
Minimum threshold	39.0	33.0	42.0	816.0	–
Points awarded	12.6	5.0	6.0	42.3	< 75.0
Maximum threshold	35.0	67.0	58.0	552.0	–
Maximum points possible	20.0	10.0	10.0	60.0	100.0

*Ab. Circ* Abdominal circumference measurement, *in* Inches

Similar to the longitudinal assessment, there was an unanticipated absence of differences between class ranks. Only the sit-ups showed significant differences; the freshman class performed significantly fewer sit-ups than the sophomore and junior classes did. Again, this result may be due to the excellent fitness status of the cadets who participated in this investigation. To the best of our knowledge, no other studies in the ROTC cadet population have examined differences between class ranks. In addition, the majority of research studies available at this present time have involved the U.S. Army ROTC population [5, 7–13]. As previously mentioned, the fitness assessments of the U.S. Army [3] and the U.S. Air Force [4] differ in the distance, time, and point allocation of the components being evaluated. Thus, only speculations can be made when comparing the two assessments.

Quantifying an individual’s physical fitness can be assessed through a variety of methods, but the methods are commonly characterized as field-based or clinical methods. While the PFA utilized by the U.S. Air Force is meant to measure airmen’s physical condition via cardiovascular endurance (a 1.5-mile run), muscular endurance (1 min of pushups and 1 min of sit-ups), and body composition (abdominal circumference); muscular strength and flexibility, which are considered basic components of physical fitness, are not measured [15]. The inclusion of muscular strength and flexibility components in the U.S. Air Force PFA may aid in a more comprehensive physical fitness evaluation. In addition, although these field-based assessments allow for time-efficient evaluations of large groups, their ability to provide accurate and discriminate analyses may be limited [5, 16]. However, clinical assessments of physical fitness may possess a higher degree of reliability and validity in comparison to field-based measurements [17]. While previous studies by Thomas et al. [5] and Oliver et al. [14] implemented both field-based (U.S. Army physical readiness test) and clinical assessments (VO_2max_, 1-repetition maximum bench press, vertical jump, body fat percentage) in their studies with U.S. Army ROTC cadets, these measures served as more of a physical fitness profile regarding the cadets’ status in training than an evaluation of the validity of the field-based assessments. Future research with ROTC cadets or tactical athletes (active duty military members, firefighters, or police officers) may consider incorporating more clinical assessments in addition to field-based assessments to provide a better understanding of the individuals’ physical performance characteristics. However, if both types of assessments are implemented, a regression between the measurements examining similar components (e.g., cardiovascular endurance: 1.5-mile run and VO_2max_) should be conducted to determine the validity of the field-based measurements.

The purpose of this investigation was to provide a longitudinal analysis of the PFA scores of U.S. Air Force ROTC cadets over a four-year period. To our knowledge, no other studies have examined the longitudinal performance of U.S. Air Force ROTC cadets in the PFA or compared their PFA scores between class ranks. There were few differences observed in the present study, which may be due to the fitness status of the participants, a relatively small number of participants, and/or the small amount of mandatory PT (2 h per week). In addition, although the cadets in this study scored well on the PFA, it is important to note that effort/motivation may have been a potential factor impacting the results. There is a possibility that once cadets reached the maximum score needed for a component, they slowed or stopped their pace, preventing them from completing as many pushups or sit-ups as possible or running as fast as possible. Nonetheless, of the components evaluated in the present study, only the sit-up component may be able to discriminate between the class ranks. These findings suggest that the U.S. Air Force PFA may not be sensitive enough to distinguish physical performance characteristics of high-performing cadets between class ranks in the ROTC population. As a result, the assessment may not be sensitive enough to identify changes across 4 years of physical training.

## Conclusions

With the exception of the sit-up component, no changes in physical performance were observed among the ROTC cadets. While the U.S. Air Force ROTC PT may not improve physical fitness (as measured by the current PFA); however, it is important to note that the PT was able to maintain the exceptionally high scores produced by the cadets who were examined in this investigation. Therefore, if the goal is the maintenance of a fitness level, then it appears that the current PT regimen is suitable. However, for cadets who are struggling to meet current PFA standards, it may be beneficial for the PT to be adjusted to improve their physical performance, increase retention rates, and better prepare them physically for their future military career. Incorporating other components of physical fitness (e.g., muscular strength and flexibility) as well as potential lab-based assessments may aid in understanding the current fitness levels of cadets and servicemembers. Implementing a more comprehensive PFA may provide more information to assist physical training leaders in building and facilitating PT regimens. These findings suggest that the current PFA may not be able to detect changes in physical fitness or distinguish between class ranks in terms of physical performance in ROTC cadets.

## Data Availability

The datasets used and/or analyzed in the current study are available from the corresponding author upon reasonable request.

## Abbreviations

AFI: Air Force Instruction
FR: Freshmen
JR: Juniors
PFA: Physical Fitness Assessment
PT: Physical Training
ROTC: Reserve Officers’ Training Corps
SO: Sophomores
SR: Seniors

## References

[1] Knapik JJ, Daniels W, Murphy M, Fitzgerald P, Drews F, Vogel J (1990). Physiological factors in infantry operations. Europ J Appl Physiol Occup Physiol.

[2] Knapik JJ, Sharp MA, Darakjy S, Jones SB, Hauret KG, Jones BH (2006). Temporal changes in the physical fitness of US Army recruits. Sports Med.

[3] U.S. Department of the Army (2013). Army Physical Readiness Training. Field Manual 7–22.

[4] Department of the Air Force (2013). Fitness Program: AFI 36-2905.

[5] Thomas DQ, Lumpp SA, Schreiber JA, Keith JA (2004). Physical fitness profile of Army ROTC cadets. J Strength Cond Res..

[6] American College of Sports Medicine Position Stand (1998). The recommended quantity and quality of exercise for developing and maintaining cardiorespiratory and muscular fitness, and flexibility in healthy adults. Med Sci Sports Exerc.

[7] Crombie AP, Lue PY, Ormsbee MJ, Llich JZ (2012). Weight and body-composition change during the college freshman year in male general-population students and Army reserve officer training corps (ROTC) cadets. Int J Sport Nutr Exerc Metab.

[8] Gist NH, Freese EC, Ryan TE, Cureton KJ (2015). Effects of low-volume, high-intensity whole-body calisthenics on Army ROTC cadets. Mil Med.

[9] Jones K, DeBeliso M, Sevene TG, Berning JM, Adams KJ (2012). Body mass index and Army physical fitness test standards in ROTC cadets. Int J Sci Eng Invest.

[10] Liguori G, Krebsbach K, Schuna J (2012). Decreases in maximal oxygen uptake among Army reserve officers’ training corps cadets following three months without mandatory physical training. Int J Exerc Sci.

[11] Schiotz MK, Potteiger JA, Huntsinger PG, Denmark DC (1998). The short-term effects of periodized and constant-intensity training on body composition, strength, and performance. J Strength Cond Res.

[12] Steed CL, Krull BR, Morgan AL, Tucker RM, Ludy MJ (2016). Relationship between body fat and physical fitness in Army ROTC cadets. Mil Med.

[13] Crawley AA, Sherman RA, Crawley WR, Cosio-Lima LM (2015). Physical fitness of police academy cadets: baseline characteristics and changes during a 16-week academy. J Strength Cond Res..

[14] Oliver JM, Stone JD, Holt C, Jenke SC, Jagim AR, Jones MT (2017). The effect of physical readiness training on reserve officers’ training corps freshman cadets. Mil Med.

[15] Heyward VH, Gibson A (2014). Advanced fitness assessment and exercise prescription.

[16] David JA, Maud PJ, Foster C (1995). Direct determination of aerobic power. Physiological assessment of human fitness.

[17] Gibbons RA, Balady GJ, Beasely JW, Bricker T, Duvernoy WFC, Froelicher DC (1997). ACC/AHA guidelines for exercise testing. J Am Coll Cardiol.

